# Hybridization-Based Detection of *Helicobacter pylori* at Human Body Temperature Using Advanced Locked Nucleic Acid (LNA) Probes

**DOI:** 10.1371/journal.pone.0081230

**Published:** 2013-11-22

**Authors:** Sílvia Fontenete, Nuno Guimarães, Marina Leite, Céu Figueiredo, Jesper Wengel, Nuno Filipe Azevedo

**Affiliations:** 1 LEPABE, Laboratory for Process Engineering, Environment, Biotechnology and Energy, Department of Chemical Engineering, Faculty of Engineering, University of Porto, Porto, Portugal; 2 IPATIMUP, Institute of Molecular Pathology and Immunology of the University of Porto, Porto, Portugal; 3 Nucleic Acid Center, Department of Physics, Chemistry and Pharmacy, University of Southern Denmark, Odense M, Denmark; 4 ICBAS, Institute of Biomedical Sciences Abel Salazar, University of Porto, Porto, Portugal; 5 FMUP, Faculty of Medicine of the University of Porto, Porto, Portugal; The University of Queensland, Australia

## Abstract

The understanding of the human microbiome and its influence upon human life has long been a subject of study. Hence, methods that allow the direct detection and visualization of microorganisms and microbial consortia (e.g. biofilms) within the human body would be invaluable. In here, we assessed the possibility of developing a variant of fluorescence in situ hybridization (FISH), named fluorescence in vivo hybridization (FIVH), for the detection of *Helicobacter pylori*. Using oligonucleotide variations comprising locked nucleic acids (LNA) and 2’-O-methyl RNAs (2’OMe) with two types of backbone linkages (phosphate or phosphorothioate), we were able to successfully identify two probes that hybridize at 37 °C with high specificity and sensitivity for *H. pylori*, both in pure cultures and in gastric biopsies. Furthermore, the use of this type of probes implied that toxic compounds typically used in FISH were either found to be unnecessary or could be replaced by a non-toxic substitute. We show here for the first time that the use of advanced LNA probes in FIVH conditions provides an accurate, simple and fast method for *H. pylori* detection and location, which could be used in the future for potential *in vivo* applications either for this microorganism or for others.

## Introduction

The human microbiome has long been studied for a better understanding of its influence upon human development, physiology, immunity, and nutrition [1]. In most of these studies, microbial identification methods rely on sample collection followed by DNA isolation and sequencing [2,3]. Despite providing important information on the communities that inhabit the human body, these methods disrupt the spatial structure of the sample, meaning that important information about human/microorganism or microorganism/microorganism interactions might be lost. In addition, the time needed to process a sample is quite long, making these methods less suitable as a diagnostic routine. Hence, novel methods which are able to address those shortcomings, by allowing the direct visualization of microorganisms and microbial consortia (e.g. biofilms) within the human body and in a short period of time, would be invaluable.

Fluorescent in situ hybridization (FISH) using DNA probes has long been used to rapidly detect and localize microbial cells in human clinical samples [4,5]. Nonetheless, this method was never employed to detect microorganisms within the human body (or other higher-order animals). The emergence of a new variant of FISH, here named as fluorescence *in vivo* hybridization of microorganisms (FIVH), has mainly been hindered by two factors. The first was the lack of suitable systems that were able to detect fluorescence signals within the human body. This issue has been recently overcome, with the arrival of medical devices with built-in advanced imaging systems, such as the confocal endomicroscope that allows an in depth analysis of the mucosa of the stomach [6] or colon [7]. So far, this device has only successfully allowed the detection of microorganisms in the human gastrointestinal-tract using non-specific staining methods [8,9]. The second factor is the lack of control over the FIVH process, as it has to be carried out under the conditions imposed by the microenvironment where the microorganism is to be found. For microorganisms present in the mucosa of the human stomach, for instance, the method would have to be carried out at 37 °C and low pH. Adding to that, DNA probes would have to resist degradation by nucleases [10]. 

The above-mentioned reasons make it very unlikely for a DNA FIVH method to work, but the evolution of nucleic acid chemistry allowed the development of chemical variations (of the nucleobase, sugar and/or phosphate backbones) of nucleic acids that can replace the DNA as a probe. In fact modified oligonucleotides, such as locked nucleic acids (LNA) or 2’-O-methyl RNA (2’OMe), have been proven to hybridize *in vivo* with native nucleic acids with low toxic effects [11–15], and are hence good candidates to develop a successful FIVH method.

**Figure 1:**
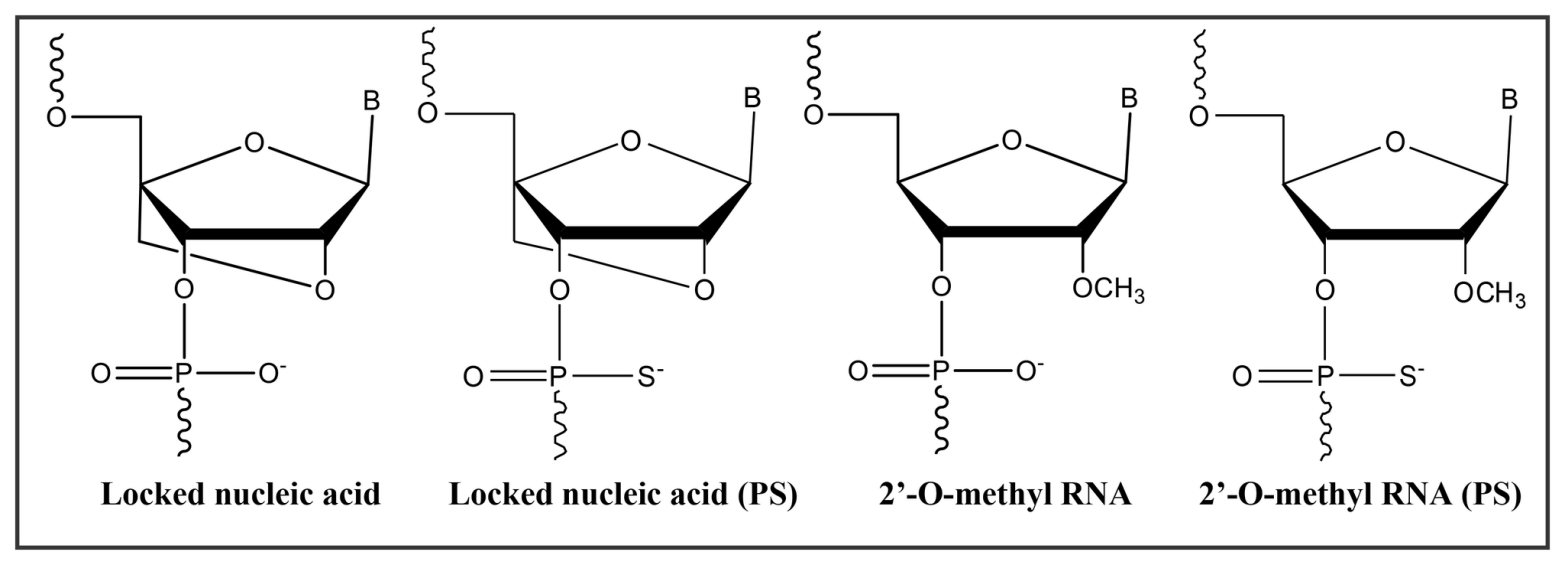
Structures of LNA and 2’ O-methyl RNA monomers (phosphate and phosphorothioate structures) used.

LNA is a nucleic acid analogue with binding sensitivity and specificity towards complementary DNA or RNA targets [16]. LNA contains a ribose ring locked by a O2’-C4’-methylene linkage resulting in a N-type (3-endo) conformation (Figure 1) [17,18]. LNA hybridizes with high affinity toward RNA (and DNA) complementary sequences according to Watson-Crick base-pairing rules, has high resistance to nuclease degradation (high bio-stability), is fully soluble in water, and display low general toxicity in animals [14,16,18]. 2’-O-Methyl-RNA based oligoribonucleotides (2’OMe) (Figure 1) constitute another nucleic acid analogue that is being used as a diagnostic probe in animal cells [19–21]. The 2’OMe group induces relatively high affinity towards an RNA target likely due to the C_3_’-endo conformation adopted by 2’OMe ribose sugars [22]. The use of 2’OMe monomers increases probe’s biostability, improves the specificity and the kinetics of hybridization, and allows targeting under conditions where DNA probes would normally not hybridize [22]. The introduction of LNA monomers into 2’OMe probes increases the target affinity even further due to an additive effect on the melting temperature (T_m_) which has been shown to improve the overall detection yield of an experiment [19,23]. 

Other types of modifications may also be incorporated to improve the target’s applicability. For instance, the use of phosphorothioate (PS) oligonucleotides presented some particularly interesting results in the case of human clinical trials as therapeutic agents for the treatment of viral infections and cancer [24,25]. The PS monomers include replacement of one of the two non-bridging oxygen atoms by a sulfur atom at each internucleotide linkage (Figure 1) [26]. These types of oligonucleotides have an increased resistance to exo- and endonucleases when compared to phosphodiester oligonucleotides (PO) [27,28], and are particularly suitable for *in vivo* applications due to their longer elimination half-life [29]. 

Taking advantage of recent progress in DNA mimics, the main objective of this work is the development of a FIVH method for the detection of microorganisms at the human body temperature (normothermia) using these novel nucleic acid molecules. As a target microorganism we selected *Helicobacter pylori* (*H. pylori*), a gram-negative and microaerophilic bacterium that colonizes the stomach of almost half of the human population and is a major risk factor associated with gastric cancer, the second deadliest cancer worldwide [30,31].

## Materials and Methods

### Bacterial strains and culture conditions

All bacterial cultures were grown in trypticase soy agar (TSA) supplemented with 5% (vol/vol) sheep blood (Becton Dickinson GmbH, Germany) and incubated for 48 hours at 37 °C under microaerobic conditions. Bacterial density was determined by the dilution of initial culture in water or saline and the absorbance was measured at 600 nm. Initial protocol optimization was performed with *H. pylori* strain 26695, obtained from the American Type Culture Collection (ATCC 700392, VA USA). For testing the specificity and sensitivity of the probes, other *H. pylori* strains and Helicobacter *spp.* were used (Table 1).

**Table 1:** Helicobacter strains tested in this study.

*Helicobacter spp.*
*H. pylori* strains
26695 (ATCC 700392)
G27 (NCTC 13282)
*H. pylori* CI-31^a^
*H. pylori* CI-116^a^
*Non-pylori Helicobacter strains*
*H. cinaedi* 33221-1.2^b^
*H. mustelae* 2H1^b^
*H. salomanis* ^b^
*H. muridarum* 2A5+^c^
*H. pametensis* ^c^
*H. bilis* ^b^
*H. canis* CIP104753^b^

^a^ - Own isolates

^b^ - Isolate provided by Francis Megraud.

^c^ - Isolate provided by Jay Solnick

### Oligonucleotide probe design

The 16S rRNA target region was selected based on a previous study [32]. The probe sequences were designed following guidelines for FISH-probes, including limitation of purine content to 60% and restriction of self-complementarity to 3 base pairs. Different types of modified nucleotide monomers were used in the probes (Figure 1). The position and the number of LNA and 2’OMe substitutions used in each probe were based on previous reports [33,34]. At the moment, a specific and reliable thermodynamic model to predict the hybridization temperature is still absent for LNA and 2’OMe-RNA. However is possible to study theoretical melting temperature of each probe through a 2’OMeRNA/Calculator online (http://rnachemlab.ibch.poznan.pl/calculator1.php). As such, we have designed several probes with different sizes to increase the chances of finding one or more probe(s) working at human body conditions (37 °C). Once the probes were selected, a search was conducted at the available 16S rRNA databases of Ribosomal Database Project II (RDP-II), version 10 (http://rdp.cme.msu.edu/) to confirm the theoretical specificity and sensitivity of the probe against other microorganisms. For this analysis, only high quality sequences with more than 1200 bp were selected [35]. It was found that all probes differed by at least two bases (two mismatches) from non-*H. pylori* species. 

### Oligonucleotides synthesis and purification

Oligonucleotides were synthesized under anhydrous conditions using standard phosphoramidite chemistry on an automated nucleic acid synthesizer (PerSpective Biosystems Expedite 8909 instrument). LNA and 2’OMe monomers were commercially available from Exiqon and Ribotask, respectively. The syntheses were performed in 1.0 µmol scale using a universal polystyrene-based support. The synthesis conditions used for the incorporation of LNA and 2’OMe monomers were as follows: trichloroacetic acid in CH_2_Cl_2_ (3:97) as detritylation reagent; 0.25 M 4,5-dicyanoimidazole (DCI) in CH_3_CN as activator; acetic anhydride in THF (9:91, v/v) as cap A solution; *N*-methylimidazole in THF (1:9, v/v) as cap B solution. As an oxidizing solution 0.02 M iodine in H_2_O/pyridine/THF was used for phosphate oligonucleotides. As a thiolation solution 0.0225 M xanthan hydrate in pyridine/CH_3_CN (20:90, v/v) was used for phosphorothioate oligonucleotides. Coupling time was 4.6 min for both monomers. Fluorescein phosphoramidite, FAM (Glen Research, VA, USA) was added in anhydrous acetonitrile (0.1 M) and activated by tetrazole with a 20 min coupling time. The stepwise coupling yields (95-99% per step) were based on the absorbance of the dimethoxytrityl cations (DMT^+^) released after each coupling step. The cleavage from the support was carried out by using 98% aqueous methanol/ammonia solution 7 N in methanol (1:1), 2 h at room temperature followed by 32% aqueous ammonia solution, 12 h at 55 °C.

All oligonucleotides were purified by reversed phase HPLC (RP-HPLC) using a Waters 600 system equipped with an XBridge OST C18 (2.5 µm, 19×100 mm) column and an XBridge Prep C18 (5 µm, 10×10 mm) precolumn. After removal of the DMT-group, oligonucleotides were characterized by ionexchange HPLC (IE-HPLC) on a Dionex system HPLC (VWR) and by matrix-assisted laser desorption ionization time-of-flight mass spectrometry (MALDI-TOF) on a Microflex Maldi (Bruker instruments, Leipzig, Germany) The purified oligonucleotides were precipitated by acetone and their purity (>90%) and compositions were verified by IE-HPLC and MALDI-TOF analysis, respectively. 

Because the probes will target the rRNA of *H. pylori*, the melting temperature of the synthetic oligonucleotides was assessed using non-modified oligonucleotides consisting only of RNA (HP_RNA_Target), fully complementary to the probes designed here (purchased from Integrated DNA Technologies). A DNA probe to serve as a reference probe (HP_DNA_Ref) with a higher number of bases than the LNA and 2’-OMe probes was purchased from Sigma-Aldrich (MO, USA).

### Melting temperature studies

From each strand, 1.0 µM was used in the following buffers: a medium salt buffer with 220 mM Na^+^ (200 mM NaCl, 20 mM NaH_2_PO_4_ and 0.2 mM EDTA, pH 7.0), a medium salt buffer with 30% (vol/vol) formamide and a low salt buffer with 30% (vol/vol) formamide with 110 mM Na^+^ (110 mM NaCl, 5 mM EDTA, 50 mM Tris-HCl and 30% (v/v) formamide, pH 7.5). After mixing each sample and denaturing the complex by heating up to 85 °C during 5 min, samples were cooled to the starting temperature of the experiment. Quartz optimal cells with a path lengh of 1.0 cm were used. Melting temperatures (T_m_ values/°C) were measured on Perkin Elmer Lambda 35 UV/VIS spectrometer equipment with a PTP 6 (Peltier Temperature Programmer) and determined as the maximum of the first derivative of the thermal denaturation curve (A_260_ vs. temperature for medium salt buffer). A temperature range from 13-15°C to 80-85 °C and a ramp of 1.0 °C/min were used. Reported T_m_ values are an average of two measurements within ± 1°C. 

### Optimization of probe hybridization conditions on slides and in suspension

The hybridization procedures developed to detect *H. pylori* were performed by FISH and evaluated by two independent techniques: by fluorescent microscopy on slides (to obtain a faster but qualitative assessment of the fluorescence signal) and by imaging flow cytometry sorting in bacterial suspensions (to obtain a quantitative assessment). Detection of 16S rRNA in slides by FISH was performed mostly as described in Azevedo et al. [36], with a few modifications. For fixation on glass slides, smears of each species/strain were immersed in 4% (v/v) paraformaldehyde for 15 min at room temperature, followed by a treatment of 50% (vol/vol) ethanol for 10 min and allowed to air dry. The hybridization was performed using 20 µl of hybridization buffer with 200 nM of the respective probe, which covered each smear individually. Two different types of hybridization buffer (pH 7.5) were tested: one containing 50% (vol/vol) formamide (Across Organic, New Jersey, US) and the other 4 M of urea (VWR BHD Prolabo, Haasrode, Belgium). The following reagents were common to both buffers: 10% (vol/vol) dextran sulphate (Fisher Scientific, MA, US), 0.1% (vol/vol) Triton-X (Panreac, Barcelona, Spain), 5 mM of EDTA disodium salt 2-hydrate (Panreac), 50 mM Tris-HCl (Fisher Scientificm New Jersey, US), 900 mM NaCl (Panreac). Samples were covered with coverslips and incubated for 90 min at 37 °C. Slides were subsequently washed in a prewarmed solution (pH 10), containing 5 mM Tris Base (Fisher Scientific), 15 mM NaCl (Panreac) and 1% Triton X (Panreac), for 30 min at 37 °C and then, the slides were allowed to air dry. All experiments were performed in triplicate and for each experiment a negative control (same hybridization conditions, but without a probe in the hybridization solution) was included. Slides were stored in the dark before microscopy analysis. For image acquisition a Leica 2000 epifluorescence microscope (Leica Microsystems GmbH, Wetzlar, Germany) was used. FAM-labeling was excited by using a 488 nm laser; the exposure time was fixed for all preparations. The fluorescence intensity of each probe and sample was quantified in the microscopy images using the ImageJ software (http://rsbweb.nih.gov/ij/index.html). 

The hybridization method in suspension was based on procedures described by Almeida et al. [37], with slight modifications. Each type of bacterium was collected from one TSA plate with 1 mL of saline and centrifuged at 14 000 x *g* for 15 min. The pellet was resuspended in 400 µL of 4% (v/v) paraformaldehyde for 1 hour, followed by centrifugation at 14 000 x *g* for 5 min. The fixed cells were resuspended in 500 µL of 50% (vol/vol) ethanol and incubated at -20°C for at least 30 min. For bacteria disaggregation the samples were subjected to sonication by ultrasounds (Transsonic 420, Elma, Germany) for 12 min, followed by a filtration through a sterile 10 μm pore-size filter (CellTrics®,Görliz, Germany). Afterwards, 100 µL of fixed cells were resuspended in 100 µL of hybridization solution (as previously described) with 400 nM of probe and incubated at 37 °C for 90 min. After hybridization the samples were centrifuged at 14,000 rpm for 5 min, resuspended in 500 µL of washing solution (as described above) and incubated at 37 °C for 30 min. The cells were again centrifuged at 14 000 x *g* for 5 min and resuspended in 100 µL of saline. To remove aggregates samples were filtered by a sterile filter with 10 μm pore size (CellTrics®). Samples were used directly for imaging flow cytometry analysis. 

### Image Quantification

Using the ImageJ program (National Institutes of Health Software), each image obtained by microscopy was analyzed for the mean fluorescence intensity. Background values were obtained by measuring a blank region from each image and these were removed from the test frames. Data was plotted as mean of arbitrary fluorescence units (AFU) which represented the mean fluorescence intensity minus background intensities. 

### Imaging flow cytometry and data analysis

*H. pylori* bacterial cell suspensions stained with FAM-labeled LNA probes, and the respective unstained negative controls, were analysed in an ImageStream^X®^ (Amnis Corporation, Seattle WA, USA) imaging flow cytometer equipped with two lasers (488 nm and 785 nm), a 40x magnification objective of 0.75 N.A, and one CDD camera. Images acquired using the INSPIRE^TM^ software included a brightfield image (Channel 1, 430-480 nm), and a green fluorescence image (Channel 2, 480-560 nm). During the sample acquisition, the area feature was used as cell classifier, in the brightfield channel, with a lower limit of 2, to exclude debris, and an upper limit of 20, to exclude bacterial aggregates and thus minimize the error in the fluorescence signal intensity that bacterial aggregates would represent in the data analysis. For each sample, 50,000 events were collected and two independent experiments were performed. All imagery data was analysed using the algorithms of IDEAS^®^ v4.0 software (Amnis Corporation). Hierarchical gating schemes were used to further eliminate bacterial aggregates and to determine the spot count and the mean fluorescence signal intensity of each bacterial sample.

### Hybridization in gastric biopsies

Formalin-fixed paraffin-embedded biopsies of gastric tissue sections from one patient infected with H. pylori were used. The use of the biopsy for research purposes was previously approved by the ethics committee of the Portuguese Institute of Oncology (IPO) in Porto, and informed written consent was obtained from the patient.Sections were cut to 3 µm thickness, and mounted on microscope glass slides and stored at 4 °C until use. Slides were immersed twice in xylol for 15 min each time, and then subjected to rehydration by decreasing concentrations of ethanol (100%, 95%, 80%, 70% and 50%) for 5 min each time. Finally, slides were washed with distilled water for 10 min and allowed to air dry. Subsequently, the hybridization procedure in slides was used as previously described using both buffers. 

### Statistical Analysis

Statistical significance was determined by One-way analysis of variance (ANOVA) by applying the Tukey multiple-comparisons test, using SPSS statistics 17.0 (SPSS, Statistical Package for the Social Sciences, Chicago, USA) or Microsoft Office Excel (Microsoft Corporation, Redmond, CA). Differences in data values were considered significant at values lower than 0.05.

## Results

### Melting temperature behaviour

The initial purpose of this work was to find a type of synthetic oligonucleotide that would be capable of efficiently hybridizing in a bacterium at human body temperature (37 °C). A first screening was performed to determine the melting temperature of 18 synthesized probes (data not shown). The hybridization temperature is not only affected by the type of probe, size and sequence, but also by the amount and type of other substances present in the hybridization solution. For this reason UV thermal denaturation studies were carried out in solutions containing not only the probes, but also different salt concentrations and a denaturing compound (formamide; FA).

After a biophysics analysis comparing melting and hybridization temperatures of the LNA probes (with different lengths), we concluded that the hybridization temperature should be between 15-30 °C lower than the melting temperature measured under similar conditions (i.e. in the presence of salt and FA) (unpublished data). Based on this criterion , four candidate probes, with melting temperatures ranging between 60-70°C were selected for further studies (Table 2). 

**Table 2:** Results of thermal denaturation experiments in different types of buffers for different types of oligoribonucleotides.

		Medium salt buffer		Medium salt buffer with FA	Low salt buffer with FA	
	Probes analyzed	Sequence	RNA complement T_m_ (°C)	RNA complement T_m_ (°C)	RNA complement T_m_ (°C)	
	Ref	5’-CTGGAGAGACTAAGCCCTCCAA-3’	64	69	68	
	HP_LNA_PO	5’-FAM GA^*L*^CT^*L*^AA^*L*^GC^*L*^CC^*L*^ -3’	78	69	67	
	HP_LNA_PS	5’-FAM G*A^L^^*^C*T^L^^*^A*A^L^^*^G*C^L^^*^C*C^*L*^ -3’	77	68	66	
	HP_LNA/2OMe_PO	5'- FAM G^L^ACT^*L*^AAG^*L*^CCC^*L*^-3’	79	66	67	
HP_ LNA/2OMe _PS	5'- FAM G^L*^ A*C*T^L^^*^A*A*G^L^^*^C*C*C^L*^-3’	78	63	66	

The DNA oligonucleotide probe reference (Ref) has the following sequence: 5’-CTGGAGAGACTAAGCCCTCCAA-3’. The RNA complementary oligonucleotide has the following sequence: 5’-UUGGAGGGCUUAGUCUCUCCAG-3’. LNA nucleotide monomers are represented with L superscript, 2’-OMe-RNA monomers in boldface letters, DNA nucleotides in capital letters, and phosphorothioate linkages by the symbol*, FAM- Fluorescein, FA- Formamide.

Thermodynamically, similar results were observed for the phosphate(HP_LNA_PO and HP_LNA/2OMe_PO) and phosphorothioate (HP_LNA_PS and HP_LNA/2OMe_PS) probes. Phosphorothioate probes (HP_LNA_PS and HP_LNA/2OMe_PS) showed only small differences (only 1°C variation in Tm values) when compared to the respective phosphate probes, with the exception of HP_LNA/2OMe_PS in the medium salt with FA. Although the number of LNA is higher in HP_LNA_PO and HP_LNA_PS probes (5 LNA monomers) than in HP_LNA/2OMe_PO and HP_LNA/2OMe_PS probes (4 LNA monomers) the only significant differences were observed in the melting temperatures when medium buffer with FA was used. It should be noted that the different salt concentration of the salt buffers did not significantly impact the Tm. 

### FISH detection of *H. pylori* by fluorescence microscopy

**Figure 2:**
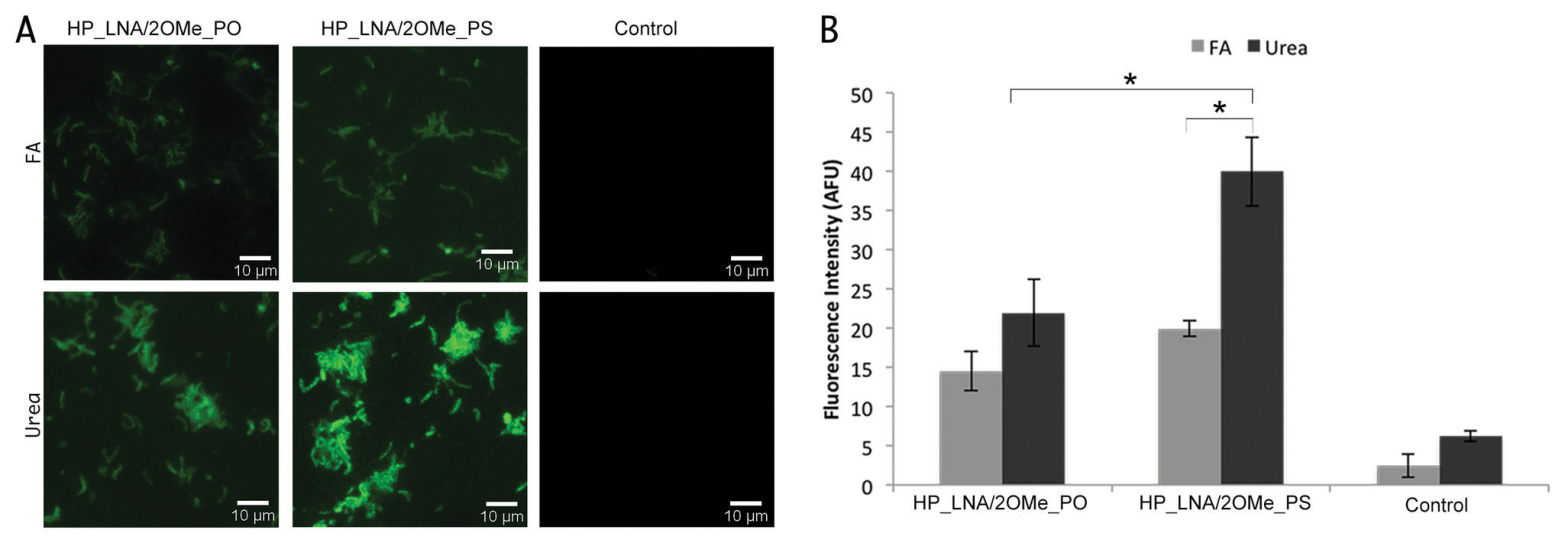
FISH detection of *H. pylori* 26695 strain (ATCC 700392) using FAM- HP_LNA/2OMe_PO and HP_LNA/2OMe_PS probes. FISH analysis was performed by epifluorescent microscopy in smears, using either 50% (vol/vol) formamide and 4 M urea as denaturing agents in the hybridization buffer. Smears without probe were used as negative control (Control) (a). (2B) Average fluorescence intensity from each probe in 4 M urea and 50% formamide (v/v) buffers; fluorescent signal intensity is expressed in arbitrary fluorescent units (AFU) and was quantified using the by ImageJ software. All images were acquired at equal exposure conditions. Original magnification: 1000x.

The ability of the selected candidate probes (Table 2) to hybridize at human body temperature (37°C) was then tested using the FISH method in glass slides. In spite of having being designed to work at similar melting temperatures, microscopy results have shown that only the HP_LNA/2OMe_PO and HP_LNA/2OMe_PS probes were able to hybridize at 37 °C (Figure 2A). Because HP_LNA_PO and HP_LNA_PS probes were only able to hybridize at temperatures higher than 40 °C (data not shown), they were not considered for further evaluation. One of the components of the hybridization solution is FA that acts as a denaturing or destabilizing agent allowing the hybridizations to be carried out at lower temperatures. However, it is not possible to use FA *in vivo* due to its toxic nature to human cells [38]. Therefore, we have tested urea at a 4 M concentration as a suitable non-toxic alternative. The hybridization efficiency of the HP_LNA/2OMe_PS probe was higher than that of the HP_LNA/2OMe_PO probe in both FA and urea buffers, as it can be observed by the high fluorescence signal (Figure 2A). Because fluorescence microscopy only provides qualitative results, we determined the average fluorescence intensity of each sample using ImageJ software (Figure 2B). 

The results obtained by ImageJ confirmed that the HP_LNA/2OMe_PS probe presents higher fluorescence intensities than the HP_LNA/2OMe_PO probe, irrespectively of the buffer used. The fluorescence intensity of the HP_LNA/2OMe_PS probe is significantly higher in the urea buffer than in the FA buffer (*p*=0.006). The difference between HP_LNA/2OMe_PS urea and the other analyzed conditions is statistically significant (*p*<0.05). No statistically significant differences were observed in fluorescence intensity between the HP_LNA/2OMe_PS probe in FA and the HP_LNA/2OMe_PO in both buffer (*p*>0.05). As expected, control experiments (without probe) showed low levels of fluorescence; this very faint background was likely due to the presence of autofluorescent substances in bacterial cells could be sometimes observed (Figure 2B).

Sensitivity and specificity of the probes are two important factors for the success of a FISH method. Because low hybridization temperatures can influence these two factors, after optimization of the hybridization conditions we have tested the sensitivity and specificity of the candidate probes against the panel of strains presented in Table 1 and in Figure S1. The candidate probes were able to detect *H. pylori* reference strains and *H. pylori* clinical isolates, while no fluorescent signal was detected for the non-pylori *Helicobacter* strains tested. These results showed that 2’-*O*-methyl/LNA-modified probes were both specific and sensitive for *H. pylori* strains, even when the hybridization is carried out at 37 °C in the presence of urea as a denaturing agent. A central parameter for a future FIVH application in the human stomach is the optimization of the FISH technique at low pH. As such, preliminary tests using HP_LNA/2OMe_PS analyzed in this study have been performed at pH 4 with positive results (data not shown). 

### FISH detection of *H.pylori* by imaging flow cytometry

**Figure 3:**
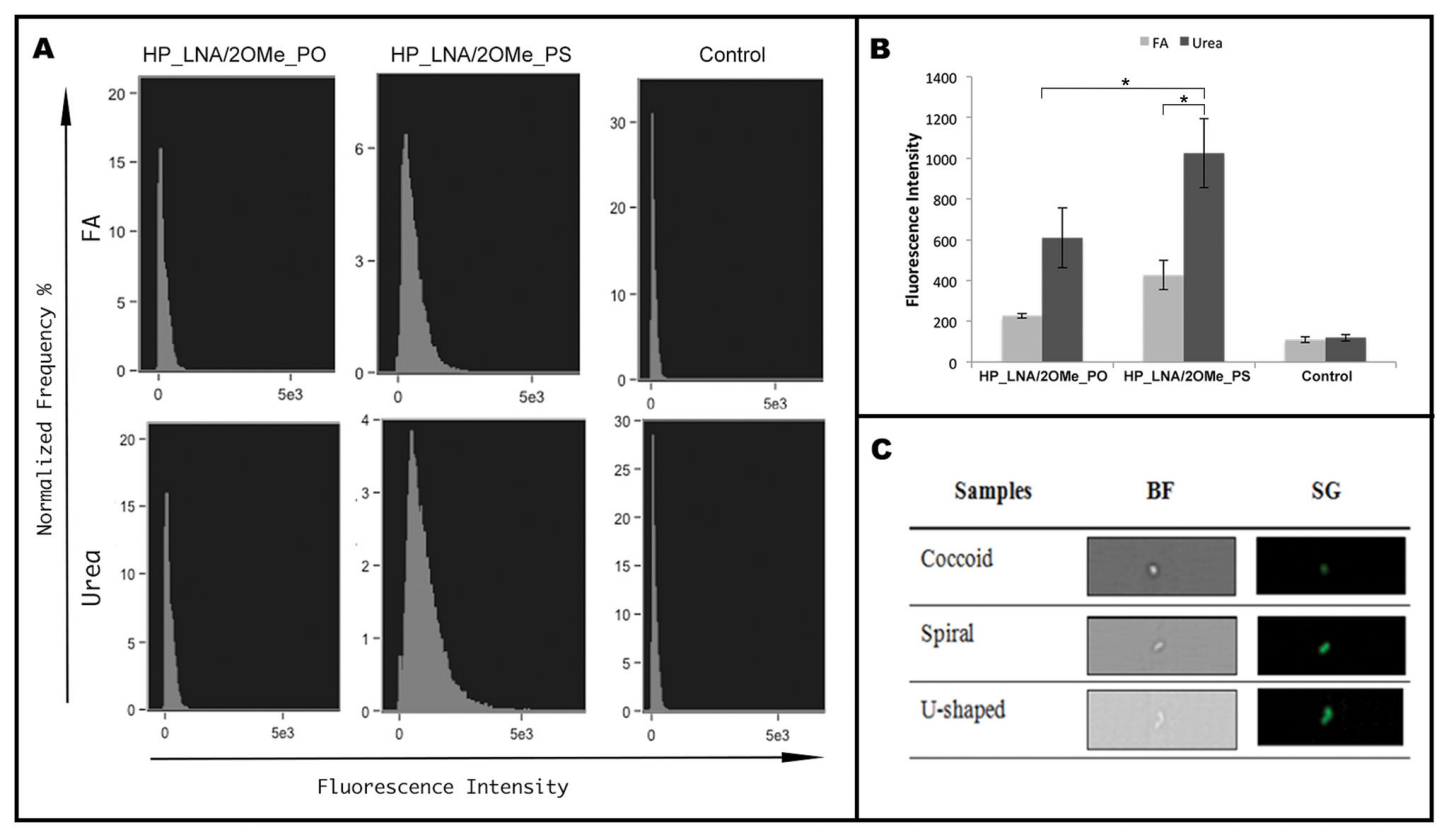
FISH detection of *H.pylori* by imaging flow cytometry. FAM labeled 2OMe/LNA probes were analysed in 50% (v/v) formamide buffer and in 4M buffer. A) Representative histograms of the green fluorescence intensity of FAM-labeled HP_LNA/2OMe_PO, HP_LNA/2OMe_PS probes and controls. B) Quantification of the mean fluorescence intensity of each probe in two independent experiments obtained by flow cytometry. C) Representative images of individual *H. pylori* with different morphologies. The population identified as individual *H. pylori* bacterium by FISH analysis was manually examined and individual cell events were identified. Individual bacterial cells, shown by Brightfield images (BF, left column) and green fluorescence images (SG, right column).

After optimization of the method on slides, we have applied the FISH procedure to *H. pylori* suspensions for imaging flow cytometry analysis. The mean fluorescence intensity of each sample was assessed and the overall results were similar to the ones determined by ImageJ for FISH application on slides (Figure 3). The HP_LNA/2OMe_PS probe in urea buffer hybridized with a significantly higher efficiency than that of HP_LNA/2Ome_PS in FA, and HP_LNA/2OMe_PO probe in both buffers (*p*<0.05), showed by the higher mean fluorescence intensity. Both probes hybridized more efficiently in the 4 M urea buffer than in the FA buffer when compared with the control without probe (Figure 3A and 3B). On the other hand, the both probes in the FA buffer did not show significant differences to the respective control (*p*>0.05) (Figure 3A and 3B). Under these conditions, using imaging flow cytometry, all different morphological types of *H. pylori* cells (spiral, coccoid and U-shaped) emitted a bright green fluorescence (Figure 3C). 

### Gastric biopsy hybridization analysis

**Figure 4:**
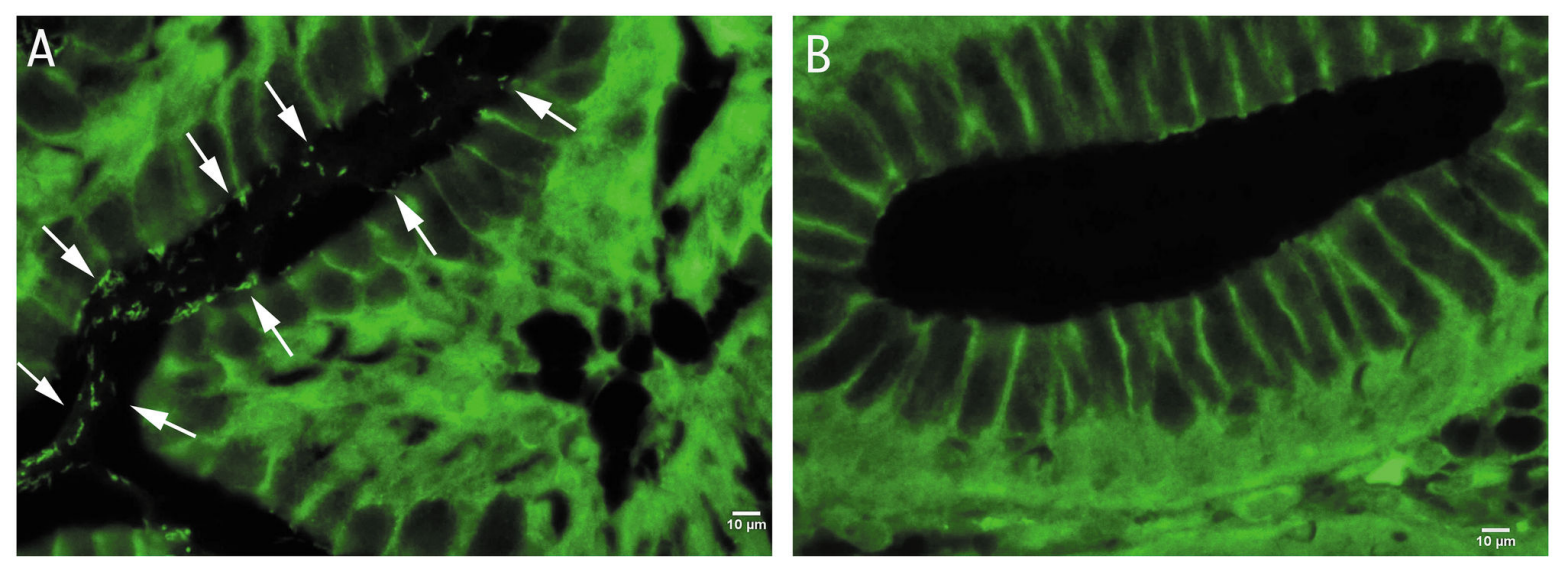
FISH detection of *H.pylori* in paraffin-embedded sections of gastric biopsies, using 2’-O-methyl/LNA FISH detection conditions. (A) Detection of *H. pylori* using the HP_LNA/2OMe_PS probe in a histological slide of a gastric biopsy specimen of an infected patient; (B) Experiment control performed in parallel using the HP_LNA/2OMe_PS probe in a histological slide of a gastric biopsy specimen of an non infected patient. Arrows indicate the presence of *H. pylori* infecting the gastric mucosa. All images were taken at equal exposure times Scale bars: 10 µm. Original magnification of ×1000.

Considering the application of 2’-O-methyl/LNA in clinical settings, the identification of *H. pylori* strains was performed in histological slides of gastric biopsy samples from patients infected with this bacterium. In all paraffin sections, bacterial rRNA was detected using FAM labeled HP_LNA/2OMe_PO and HP_LNA/2OMe_PS probes. However, the analysis of gastric biopsies using HP_LNA/2OMe_PS showed more fluorescence intensity of *H. pylori* than in the same conditions with HP_LNA/2OMe_PO (data not shown). Negative controls confirmed the lack of autofluorescence from non-labeled *H. pylori* cells (Figure 4B). Therefore, using hybridization conditions similar to FIVH it is possible to identify and locate the bacteria in the gastric mucosal surface.

## Discussion

This work presents the first approach to obtain a method for the detection of *H. pylori* under *in vivo* mimicking conditions. Taking advantage of the evolution of nucleic acid chemistry, we have synthesized a set of LNA and/or 2’-O-Methyl RNA probes using the standard phosphodiester and the synthetic phosphorothiate backbones and have evaluated their suitability to hybridize at 37 °C. 

The hybridization assays showed that only HP_LNA/2OMe_PS and HP_LNA/2OMe_PO probes were able to emit fluorescence at 37 °C. These results suggest that the substitution of DNA for 2’-O-Methyl-RNA nucleotides in LNA probes allows the hybridization to occur at lower temperatures, as long as a shorter LNA/2’-OMe-RNA sequence is used when compared to the corresponding DNA probe. It is furthermore expected that the introduction of 2’OMe monomers into LNA probes will increase probe’s biostability, specificity and kinetics of hybridization, and allows targeting under conditions where DNA/LNA probes do not hybridize, an observation that has been corroborated by others [19,22,23]. We have shown that both HP_LNA/2OMe_PS and HP_LNA/2OMe_PO probes are able to successfully hybridize with *H. pylori* RNA at 37 °C. The HP_LNA/2OMe_PS probe yield higher fluorescence intensities in slides and in suspensions of *H. pylori*, as well as in the gastric biopsies, which appears to indicate that phosphorothioate probes are the best candidates to be used as probes in this type of experiments. 

Taking in mind the future application of this method to *in vivo* conditions, and since the FISH procedure includes solutions that are toxic, we have successfully replaced FA by urea. In fact, FA, used as a destabilizing agent of nucleic acid duplexes, is one of the most hazardous chemicals present in the hybridization solution and the use of urea as a substitute has already been suggested by other authors [38]. The replacement of the common 50% (v/v) FA by 4M urea led to a 40% increase in the signal intensity, in both probes analysed (Figure 3), which could be explained by the additional permeabilization role of urea [39]. More importantly, the use of warmed urea-containing buffer did not affect the renaturation kinetics of the reaction. 

Although different studies have performed the hybridization step of a FISH process at 37 °C, these typically use a previous denaturation step and a washing step performed at higher temperatures [11,40] or long periods of incubation [41]. Furthermore, some of these studies used DNA probes, and as such the hybridization step alone lasted at least for 8 hours, which is undesirable for the final purpose of FIVH. Furthermore, these experiments have only been performed in animal cells, where nucleic acids are able to diffuse more freely into the cell due to the lack of a cell wall. The present study is the first to perform the detection of a bacterium by FISH at human body temperature used for all steps of the hybridization procedure. The only study that used the designation of FIVH and that was performed in live animal cells [42], applies hybridization in an *ex vivo* culture environment and as such is not a true FIVH. To the best of our knowledge, the few studies that were performed *in vivo* in animal cells used fluorescently-labelled small peptides as probes instead of nucleic acids [7]. Another issue observed in this study was the autofluorescence of the tissues in the biopsy. Non-specific fluorescence agents, such as fluorescein, have been used in several studies with endomicroscopy analysis [43]. The sensitivity of this technique allowed for the visualization of small cellular structures such as capillaries and inflammatory cells [44]. Therefore, although autofluorescence is present in the analyzed tissues (Figure 4), published works showed that it is possible to discriminate small fluorescence signals *in vivo* [45,46]. There are also imaging analysis methods which allow the reduction of autofluorescent signatures from image data mathematically [47,48]. Another possible approach is the use of a different exogenous fluorophores with a different spectral region where tissue autofluorescence cannot be observed [49]. Therefore, if the presence of autofluorescence in fluorescent images becomes a problem in *in vivo* analysis, there still exist different types of strategies that might allow to overcome this issue.

While significant steps have been taken in here to successfully achieve FIVH in the future, there is still much work to be carried out. For instance, other components employed in the hybridization solutions, such as dextran sulphate and Triton X will have to be substituted or have their concentrations decreased for FIVH application due to their toxicity [50,51]. Dextran sulphate is used as an hybridization rate accelerator [52], whereas Triton X acts as a detergent and prevents non-specific binding. A balance in the concentration of these reagents simultaneously with the concentration of added urea that will ensure reasonable kinetics and specificity of hybridization while keeping acceptable levels of toxicity will have to be accomplished. The exposure time of the probe to the respective target is also one of the most important factors for FIVH success. In fact, the decrease of the time-course of each experiment (30 min) has already been done in our lab for PNA probes (data not shown). Matthiesen and Hansen also tried to reduce the required hybridization time using DNA probes, however, the best results were observed only with one hour of hybridization [53]. Therefore, future studies will be also focused on decreasing the exposure time of the HP_LNA/2OMe_PS probe. 

A suitable detection system that is able to detect the fluorescence signal inside the human body to assess the efficiency of hybridization is available to FIVH, using a computer connected to a confocal laser endomicroscope [43]. This equipment has been shown to be useful for *in vivo* diagnosis of precancerous conditions and gastric cancer, and has also allowed direct, nonspecific *in vivo* identification of *H. pylori* [54]. Therefore, it could be used to detect the fluorescence signal of the HP_LNA/2OMe_PS probe *in vivo*, possibly allowing acquisition of real time high resolution images of this bacterium during ongoing endoscopy. Another important advantage of this method is that it should be easily adapted to detect the resistance of *H. pylori* to clarithromycin, by a simple redesign of the probes [55]. This would imply that by the end of an endomicroscopy, the gastroenterologist would not only know about the presence of *H. pylori*, but also have information about the best therapy that should be prescribed to the patient.

In this study, the fluorescence intensity was used as a parameter of probe hybridization efficiency in slides and in suspension. The quantification of fluorescence was performed both by the ImageJ software and by flow cytometry. One of the reasons why the quantification of fluorescence was performed by two methods was that when using flow cytometry it is possible to analyze each cell as an independent observation and therefore stronger statistical data is obtained (Figure 3). The other reason was that, within the human body, not all the microbial cells are adhered to a surface. For instance, when microbial infections of the blood occur, microorganisms such as *Candida* spp., *Staphylococcus aureus* and many others can be found in the bloodstream [56,57]. Because one of the goals of this study was to establish a framework for other FIVH methods, we considered it relevant to assess if differences in the hybridization performance could also be observed between hybridization in slides and in suspension. As this was not the case, FIVH might also be applicable in the future as a diagnostic method to detect the causative agent of a septicaemia, pending on the development of suitable technologies to detect the fluorescent signal of the probe. 

## Conclusions

Specific and sensitive detection by FISH of a microorganism under human normothermia conditions is reported herein for the first time. More importantly, the study also lays the foundation for other projects that aim to develop methods for *in vivo* detection of other microorganisms using FIVH. For instance, the remarkable properties of LNA in terms of hybridization affinity and specificity were essential for the obtained results. Furthermore, phosphorothioate internucleoside linkages coupled with the introduction of 2’OMe residues proved to be the most suitable probes. As the FIVH process is mostly controlled by the thermodynamics of hybridization of nucleic acids, it is suggested that works targeting other microorganisms should employ LNA/2OMe with PS linkage-based probes.

Future research should be focussed in three main directions: 1) decrease of the time of a standard FISH procedure; 2) evaluation of the cytotoxicity of all compounds used in this process, and 3) assessment of the ability of confocal endomicroscopy to detect the fluorescent signal emitted by the fluorochrome attached to the cells through *in vivo* studies. 

## Supporting Information

Figure S1 **Fluorescence intensity results of Helicobacter strains (non 26695 (ATCC 700392)) tested in this study.** The results represent the positive signal of each sample after the subtraction of the respective control.
(DOCX)
