# Supplementary material for: Hybridization-Based Detection of Helicobacter pylori at Human Body Temperature Using Advanced Locked Nucleic Acid (LNA) Probes
Source: PLoS One. 2013 Nov 22;8(11):e81230. doi: 10.1371/journal.pone.0081230 (PMC3838382; doi:10.1371/journal.pone.0081230)
Supplement: Figure S1 — Fluorescence intensity results of Helicobacter strains (non 26695 (ATCC 700392)) tested in this study. The results represent the positive signal of each sample after the subtraction of the respective control. (DOCX) [file pone.0081230.s001.docx]

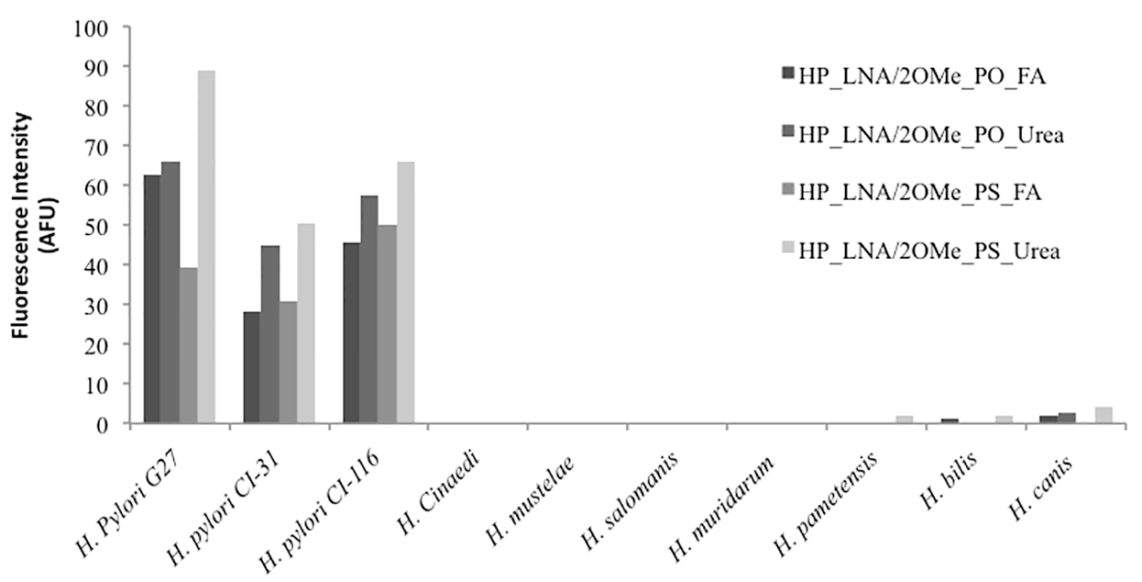


Figure S1- Fluorescence intensity results of Helicobacter strains (non 26695 (ATCC 700392)) tested in this study. The results represent the positive signal of each sample after the subtraction of the respective control.
